# Preoperative gut microbiota depletion and metabolomic signatures predict postoperative pneumonia in patients with intracranial space-occupying lesions: a multi-omics prospective cohort study

**DOI:** 10.3389/fimmu.2026.1839465

**Published:** 2026-06-16

**Authors:** Juan Chen, Zhiyu Li, Yewen Zhan, Qianqian Tan, Jin Li, Hong Pu

**Affiliations:** 1 Department of Critical Care Medicine, West China School of Medicine, West China Hospital of Sichuan University, Chengdu, China; 2 Department of Technology, Puluo (Wuhan) Medical Biotechnology Co., Ltd., Wuhan, China; 3 Department of Neurosurgery, West China School of Medicine, West China Hospital of Sichuan University, Chengdu, China

**Keywords:** early precision intervention, gut microbiota, metabolic signature, postoperative pneumonia, untargeted metabolomics

## Abstract

**Background:**

Patients with intracranial space-occupying lesions are highly susceptible to postoperative pneumonia (POP) due to extensive surgical trauma, impaired consciousness, and prolonged immobilization, which severely compromise recovery and increase mortality. While the “gut-lung axis” highlights the role of gut microbiota in distal pulmonary immune regulation, how preoperative gut signatures predict neurosurgical infections remains poorly understood.

**Methods:**

This prospective study enrolled 50 patients, collecting preoperative and postoperative fecal and plasma samples. 16S rDNA sequencing was used to monitor microbial evolution, and LC-MS/MS-based untargeted metabolomics characterized systemic metabolic profiles.

**Results:**

Although surgical stress reduced microbial diversity across all groups, the POP group exhibited significant preoperative depletion of *Roseburia*. Metabolomics revealed a preoperative “metabolic deficiency” state in POP patients, primarily involving impaired fructose and mannose metabolism. Multi-omics integration confirmed that preoperative *Roseburia* abundance positively correlated with the anti-inflammatory metabolite 1-methylnicotinamide. A metabolic signature composed of the top 10 preoperative metabolites predicted POP with an AUC of 1.0.

**Conclusion:**

The disruption of metabolic homeostasis driven by preoperative gut microbiota depletion is a key factor associated with POP susceptibility. Monitoring the preoperative metabolic signature enables precise identification of high-risk individuals and offers a novel pathway for early intervention via the gut-lung axis.

## 1 Introduction

In the field of neurosurgery, postoperative pneumonia (POP) represents a formidable clinical challenge for patients with intracranial space-occupying lesions. Due to the extensive nature of surgical trauma, fluctuations in postoperative consciousness, and the prevalent chronic wasting state of these patients, the risk of developing pulmonary infections is significantly elevated (1). Existing evidence underscores that POP not only markedly extends hospital stays but is also independently associated with unplanned intensive care unit (ICU) admissions and increased postoperative mortality, thereby severely compromising patient prognosis (2). Despite its impact, current clinical risk assessments remain heavily reliant on intraoperative variables or post-facto clinical manifestations, leaving a critical gap in pre-warning strategies capable of identifying high-risk individuals during the preoperative phase.

Recently, the emergence of the “gut-lung axis” theory has offered a novel paradigm for understanding susceptibility to postoperative infections (3, 4). An intracranial space-occupying lesion is not merely a localized anatomical mass; it often triggers a systemic stress response that induces systemic immunosuppression and potentially disrupts gut microbial homeostasis prior to intervention (5). We hypothesize that these patients may harbor a “hidden fragility” characterized by microbial depletion and insufficient metabolic reserve before undergoing surgical stress. This preexisting state may impair distal pulmonary immune defense, rendering the host highly susceptible to POP.

Gut microbiota and their functional metabolites, such as short-chain fatty acids (SCFAs) and amino acids, serve as vital “barometers” of host immune homeostasis (6, 7). Previous studies have suggested that a depletion of butyrate-producing bacteria (e.g., *Roseburia*) or a decline in circulating branched-chain amino acids (BCAAs) is directly linked to an impaired anti-inflammatory capacity (8, 9). By integrating 16S rDNA sequencing with untargeted metabolomics, it is possible to capture subtle preoperative shifts in microbial and metabolic profiles, potentially allowing for the establishment of a precision risk-stratification system before infection occurs.

In this prospective cohort study, we aim to systematically characterize the preoperative and postoperative gut microbiota and metabolic signatures in patients with intracranial space-occupying lesions. By identifying core microbial genera and metabolic biomarkers with high discriminatory power, we seek to construct a predictive model for POP. This work intends to elucidate the mechanistic link between preoperative intestinal “exhaustion” and pulmonary susceptibility, ultimately providing neurosurgeons with a robust tool for preoperative risk stratification and personalized management.

## 2 Materials and methods

### 2.1 Study population and grouping

This prospective cohort study included adult patients with intracranial space-occupying lesions who underwent surgical treatment at West China Hospital, Sichuan University between May 2024 and October 2025. The inclusion criteria were as follows: (1) age > 18 years; (2) diagnosis of intracranial space-occupying lesions confirmed by magnetic resonance imaging (MRI) or computed tomography (CT); (3) underwent craniotomy for tumor resection; and (4) availability of complete clinical data. The exclusion criteria included: (1) history of chronic intestinal diseases (e.g., inflammatory bowel disease) before surgery; (2) use of antibiotics, probiotics, or immunosuppressants within 1–3 months prior to surgery; and (3) evidence of infection in other sites before surgery. Patients were divided into an infection group and a non-infection group based on the occurrence of postoperative pulmonary infection. Postoperative pulmonary infection was diagnosed according to the Clinical Pulmonary Infection Score (CPIS) (10), characterized by clinical manifestations such as fever, purulent sputum, and new infiltrates on chest radiography. The study protocol was approved by the Ethics Committee of West China Hospital, Sichuan University (Approval No. 2024 (890)). Written informed consent was obtained from all included patients or their legal guardians regarding participation in the study and sample collection.

### 2.2 Clinical data collection

Comprehensive demographic and clinical characteristics were collected from electronic medical records for all participants. The collected baseline data included gender, age, and body weight. Disease-specific information included tumor type (glioma or meningioma), World Health Organization (WHO) tumor grade, duration of surgery, and hormone dosage.

### 2.3 Sample collection and preparation

A total of 50 patients with intracranial tumors were enrolled in this study. Paired fecal and plasma samples were collected at two time points: preoperative (within 48 hours before surgery) and postoperative (3–5 days after surgery). Fresh fecal samples (approximately 200 mg) were collected using sterile fecal collection tubes. Peripheral blood samples (3–5 mL) were collected into EDTA-coated tubes and centrifuged at 3000 rpm for 10 minutes to separate the plasma. All samples were immediately aliquoted and stored at -80°C until further analysis. Fecal samples were subsequently used for 16S rDNA gene sequencing to analyze gut microbiota diversity, while plasma samples were subjected to untargeted metabolomics analysis.

Based on the occurrence of postoperative pneumonia, the patients were divided into the postoperative pneumonia (POP) group and the postoperative non-pneumonia (Non-POP) group. According to the sampling time (preoperative and postoperative), the POP group was further subdivided into the POP-Pre subgroup and the POP-Post subgroup; the Non-POP group was further subdivided into the Non-POP-Pre subgroup and the Non-POP-Post subgroup.

### 2.4 16S rDNA gene sequencing and bioinformatics analysis

Genomic DNA was extracted from samples using the OMEGA Mag-bind Soil DNA Kit according to the manufacturer’s instructions. After quality inspection, the V3-V4 hypervariable regions of the 16S rDNA gene were amplified using specific primers with unique barcodes. PCR products were purified, quantified, and pooled to generate sequencing libraries using the Illumina TruSeq Nano DNA LT Library Prep Kit. Sequencing was performed on an Illumina platform. Raw data were processed using Cutadapt to remove primer sequences. Quality control, denoising, and chimera removal were conducted using the QIIME 2 pipeline and the DADA2 algorithm to generate an Amplicon Sequence Variant (ASV) feature table. Representative sequences were annotated against the Silva database (v138). Alpha and beta diversity indices were calculated using QIIME 2. Linear discriminant analysis Effect Size (LEfSe) (LDA score > 2, *p* < 0.05) and STAMP software were employed to identify significantly different bacterial genera between groups.

### 2.5 Untargeted metabolomics detection and analysis

Metabolites were extracted from samples using a methanol/acetonitrile/water (2:2:1, v/v/v) mixture. The supernatant was collected after centrifugation, vacuum-dried, and reconstituted. Chromatographic separation was performed on a Vanquish UHPLC system (Thermo Fisher Scientific) equipped with a HILIC column. Mass spectrometry data were acquired using an Orbitrap Exploris™ 480 mass spectrometer in both positive and negative electrospray ionization (ESI) modes. The detection process is performed continuously in batches. Quality control (QC) samples are prepared by mixing equal volumes of the extraction solutions from the test samples and are analyzed before, during, and after LC-MS/MS injection of the test samples. Experimental data quality is evaluated through six QC parameters: Total Ion Current analysis of QC samples, Principal Component Analysis of the overall sample data, correlation analysis of QC samples, Hotelling’s T2 test for the overall sample, Multivariate Control Chart analysis of QC samples, and Relative Standard Deviation analysis of QC samples. Raw data files were converted to.mzXML format using ProteoWizard and processed with XCMS software for peak alignment and extraction. Metabolite identification was performed by matching exact molecular masses (< 10 ppm) and MS/MS spectra with an in-house standard database (Shanghai Applied Protein Technology). Multivariate statistical analyses, including PLS-DA and OPLS-DA, were conducted to evaluate group differences. The Benjamini-Hochberg (BH) procedure was applied to adjust p-values for multiple testing, controlling the false discovery rate (FDR). Differential metabolites were identified based on Variable Importance in Projection (VIP) > 1 and Student’s *t*-test *p* < 0.05.

### 2.6 Statistical analysis

Statistical analyses were performed using SPSS software (version 19.0, IBM Corp., Armonk, NY, USA) and R packages on Mac OS X. Continuous variables were first tested for normality using the Shapiro-Wilk test. Normally distributed data were expressed as mean ± standard deviation (SD) and compared using the Student’s t-test, while non-normally distributed data were presented as median (interquartile range, IQR) and compared using the Mann-Whitney U test. Categorical variables were expressed as frequencies (percentages) and compared using the Chi-square test or Fisher’s exact test. For multi-omics association analysis, Spearman correlation coefficients were calculated to evaluate the relationships between significantly differential bacterial genera (LEfSe LDA > 2, *p* < 0.05) and differential metabolites (VIP > 1, *p* < 0.05). Hierarchical clustering heatmaps were generated using the ‘pheatmap’ package in R, and correlation networks were constructed using Cytoscape software (v3.5.1) to visualize key interactions, with a threshold of absolute correlation coefficient |r| > 0.5 and *p* < 0.05. Additionally, metabolic pathway enrichment analysis was performed by mapping differential metabolites to the Kyoto Encyclopedia of Genes and Genomes (KEGG) database (https://www.kegg.jp/). The enrichment significance was evaluated using a hypergeometric test, and key biological pathways were identified based on the Rich Factor and -*log*_10_(*p*-value). Furthermore, Receiver Operating Characteristic (ROC) curve analysis was conducted to assess the predictive value of key differential metabolites and/or bacterial genera for postoperative pulmonary infection. The Area Under the Curve (AUC) and its 95% Confidence Interval (CI) were calculated to quantify predictive accuracy. All statistical tests were two-tailed, and a *p* < 0.05 was considered statistically significant.

## 3 Results

### 3.1 Characteristics of the study population

**Figure 1:**
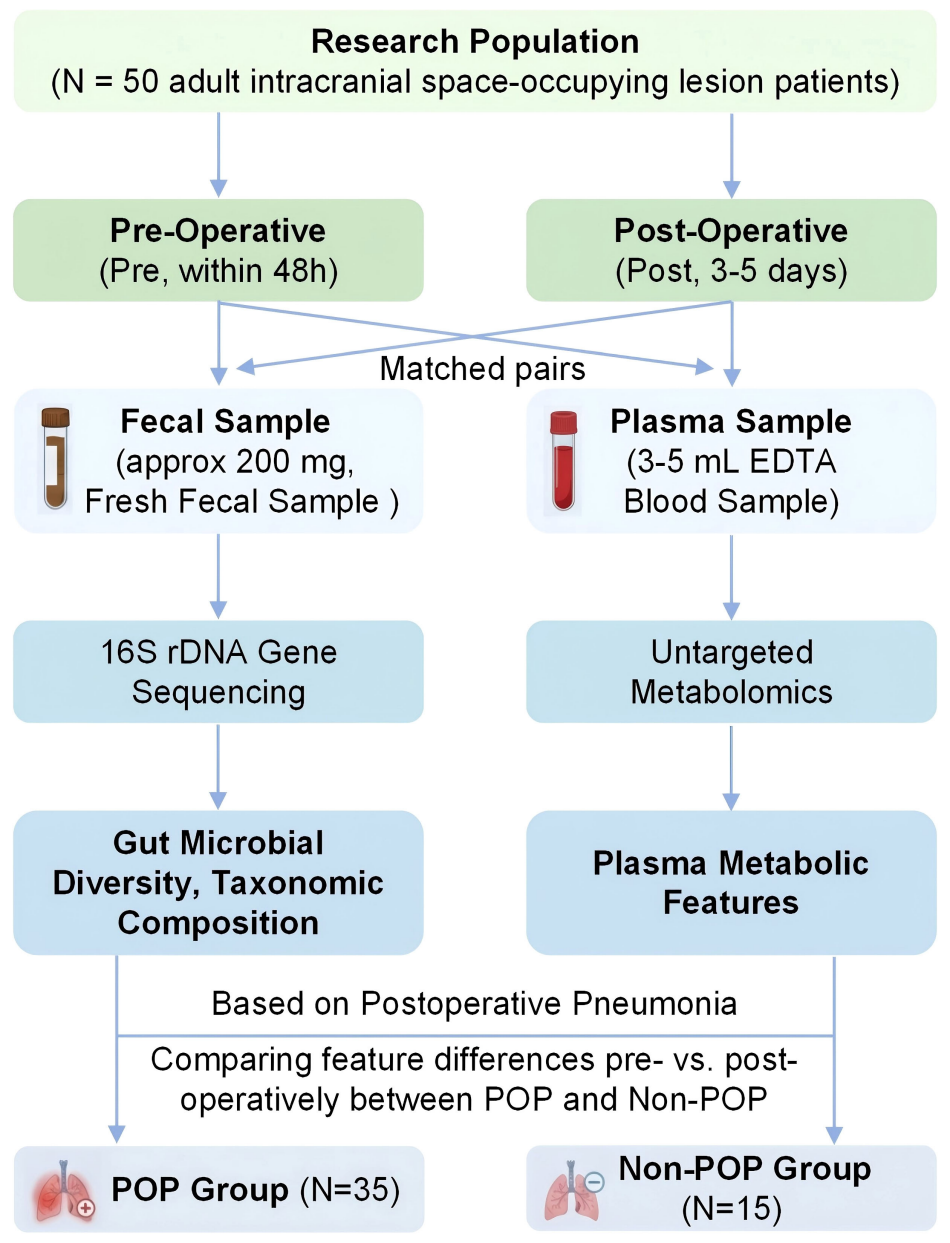
Flowchart of the research design. POP Group, Postoperative Pneumonia Group; Non-POP Group, Non-postoperative Pneumonia Group.

We conducted a prospective cohort study to investigate the dynamic changes in gut microbial and metabolic characteristics among adult patients undergoing surgery for intracranial space-occupying lesions, comparing preoperative baselines with postoperative outcomes (**Figure 1**). A total of 50 patients were enrolled, including 35 in the postoperative pneumonia (POP) group and 15 in the non-pneumonia (Non-POP) group. No statistically significant differences were observed between the two groups in terms of age, gender, BMI, height, weight, smoking history, surgical duration, ICU admission duration, or ventilator use duration (**Supplementary Table 1**). All enrolled patients were in good nutritional condition. Furthermore, there were no significant differences in comorbidities between the two groups (*p* = 0.533), including hypertension, history of other tumor resections, and previous surgery for cerebral hemorrhage. Tumor types were diverse (including meningiomas, schwannomas, etc.), but no significant bias was found in tumor WHO grading between the two groups (*p* = 0.794). Both groups received routine antibiotics during surgery, primarily cefazolin sodium 2 g. Postoperative therapeutic antibiotics were administered more frequently in the POP group (65.7% vs. 26.7%, *p* = 0.015), consistent with the clinical requirement for treating confirmed infections. The diagnostic time for POP was 16.73 ± 5.48 hours, and all postoperative samples in the POP group were collected after the onset of pneumonia infection.

### 3.2 Spatiotemporal evolution of gut microbiota

**Figure 2:**
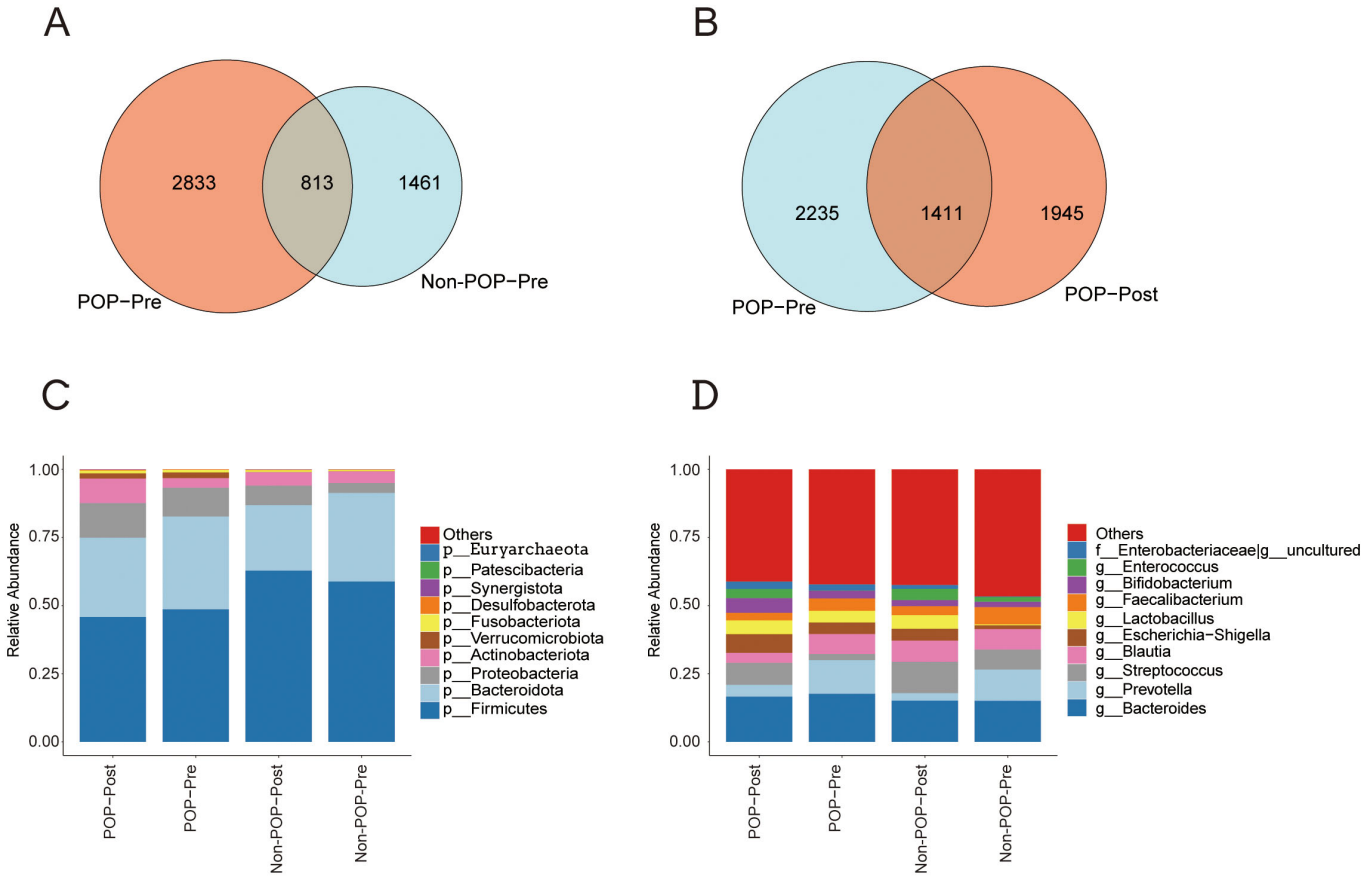
Characteristics of gut microbiota composition before and after surgery. **(A, B)** Venn diagrams of ASVs across subgroups. **(C, D)** Relative abundance of bacterial communities at the phylum **(C)** and genus **(D)** levels. POP-Pre, preoperative of postoperative pneumonia; POP-Post, postoperative of postoperative pneumonia; Non-POP-Pre, preoperative of postoperative non-pneumonia; POP-Post, postoperative of postoperative non-pneumonia. Other items confirmed as correct.

Fecal samples collected perioperatively from the 50 patients were analyzed using 16S rDNA sequencing, yielding 59,928 ± 7,39 reads per sample. Clustering analysis identified 8,630 Amplicon Sequence Variants (ASVs). A comparison between the POP-Pre and Non-POP-Pre subgroups showed 813 shared ASVs, with 2,833 and 1,461 ASVs unique to each subgroup, respectively (**Figure 2A**). Between the POP-Post and POP-Pre subgroups, 1,411 ASVs were shared, while 2,235 and 1,945 ASVs were unique to each (**Figure 2B**). Preoperatively, ASV levels differed markedly between the POP and Non-POP groups. **Figure 2C** illustrates the top 10 most abundant bacterial phyla, primarily comprising *Firmicutes*, *Bacteroidota*, *Proteobacteria*, *Actinobacteriota*, *Verrucomicrobiota*, and *Fusobacteriota*. At the genus level, the community was dominated by *Bacteroides*, *Prevotella*, *Streptococcus*, *Blautia*, *Escherichia-Shigella*, and *Lactobacillus* (**Figure 2D**).

**Figure 3:**
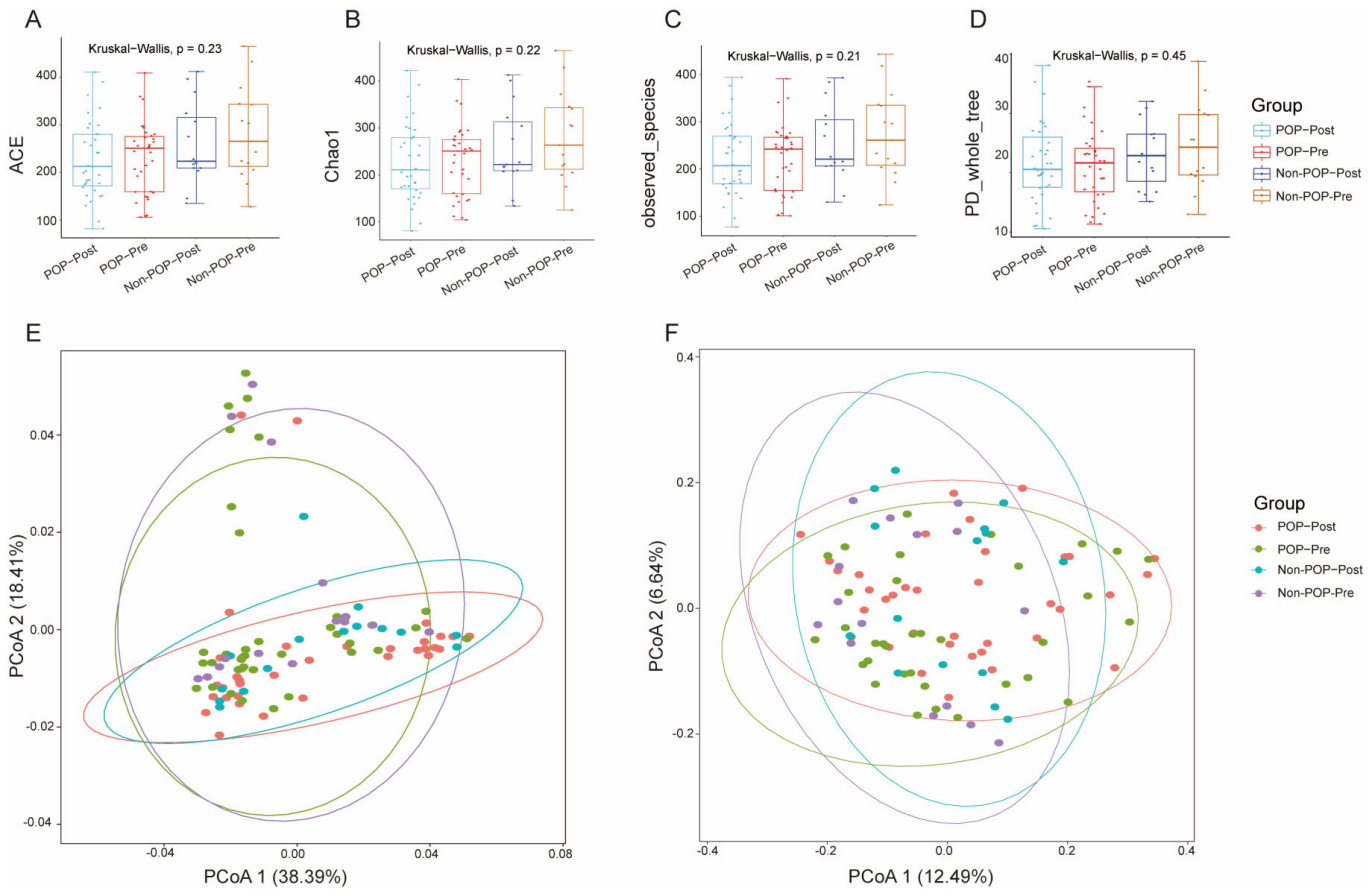
Analysis of gut microbiota diversity before and after surgery. **(A–D)** α-diversity indices of gut microbiota among the four subgroups: ACE **(A)**, Chao1 **(B)**, Observed_species **(C)**, and PD_whole_tree **(D)**. **(E, F)** Principal Coordinate Analysis (PCoA) of β-diversity based on Weighted **(E)** and Unweighted **(F)** UniFrac distances. POP-Pre, preoperative of postoperative pneumonia; POP-Post, postoperative of postoperative pneumonia; Non-POP-Pre, preoperative of postoperative non-pneumonia; POP-Post, postoperative of postoperative non-pneumonia.

α-diversity analysis, used to assess within-community microbial diversity, showed no significant differences between groups; however, diversity indices were lower in the postoperative groups compared to preoperative groups, and lower in the POP group compared to the Non-POP group (**Figures 3A–D**). β-diversity analysis based on Weighted and Unweighted UniFrac distances revealed significant differences between the POP-Post and POP-Pre subgroups (Anosim: *R* = 0.047, *p* = 0.027 and *R* = 0.055, *p* = 0.010; Adonis: *R^2^* = 0.025, *p* = 0.018 and *R^2^* = 0.040, *p* = 0.018). The principal component analysis (PCoA) results are shown in **Figures 3E, F**.

### 3.3 Preoperative bacterial signature

**Figure 4:**
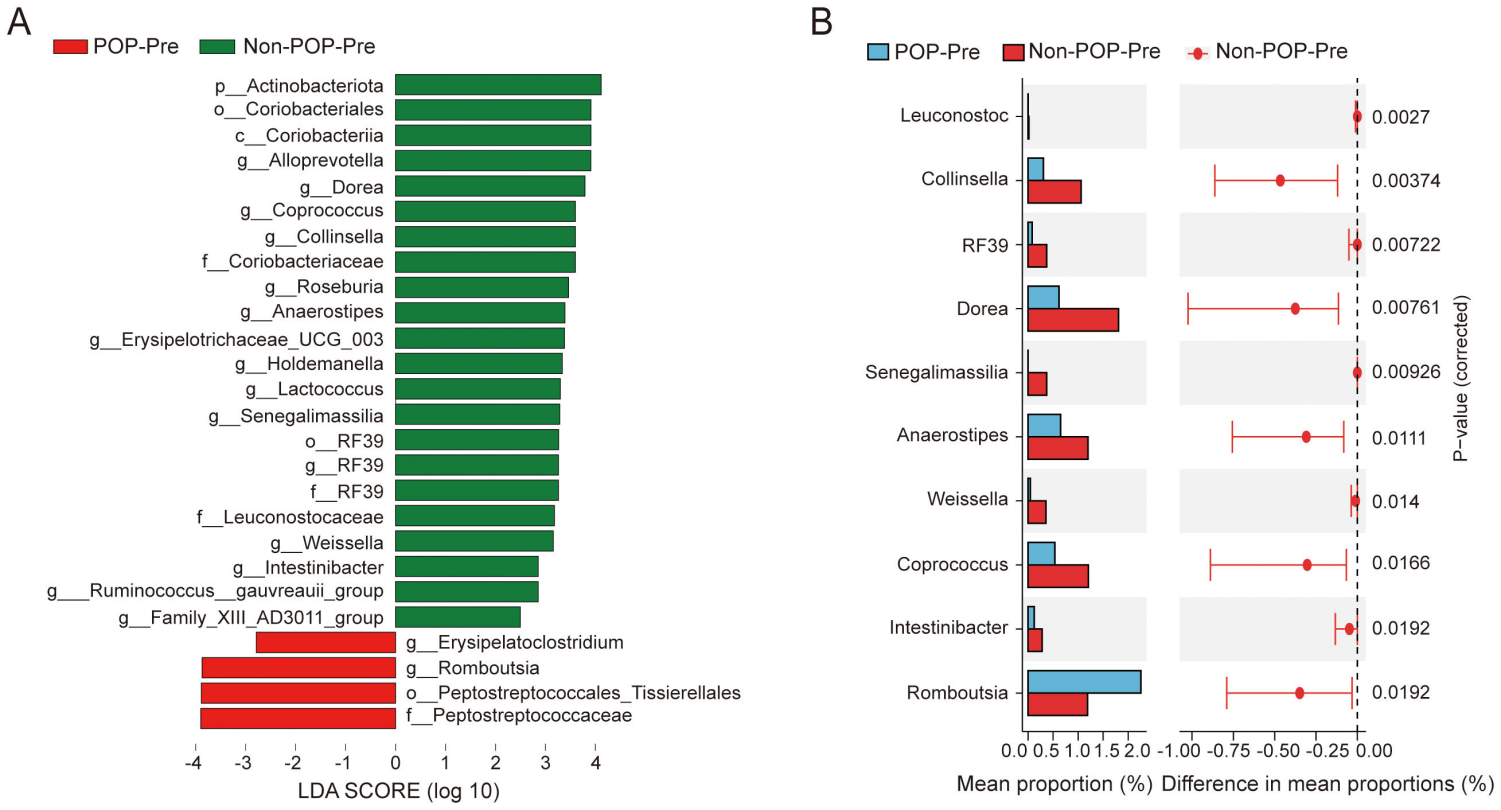
LEfSe and STAMP analysis of gut microbiota between POP-pre and non-POP-pre subgroups. **(A)** LEfSe analysis identifying differentially abundant taxa (LDA Score > 2.0). **(B)** STAMP analysis showing significant differences in mean proportions of microbial genera between subgroups. POP-Pre, preoperative of postoperative pneumonia; Non-POP-Pre, preoperative of postoperative non-pneumonia.

LEfSe analysis revealed that the Non-POP-Pre subgroup, compared to the POP-Pre subgroup, exhibited a gut microbiota composition that included key taxa such as *Actinobacteriota* and *Coriobacteriaceae*. At the genus level, *Roseburia*, *Coprococcus*, *Collinsella*, and *Lactococcus* exhibited higher relative abundances in this subgroup. In contrast, *Romboutsia* and *Erysipelatoclostridium* were enriched in the POP-Pre subgroup (**Figure 4A**). STAMP analysis further confirmed that the Non-POP-Pre subgroup possessed a more robust composition of butyrate-producing bacteria, including significantly upregulated *Coprococcus* and *Anaerostipes*. The POP-Pre subgroup was primarily characterized by the significant enrichment of *Romboutsia* (**Figure 4B**).

### 3.4 Metabolic deficiency and pathway alterations

**Figure 5:**
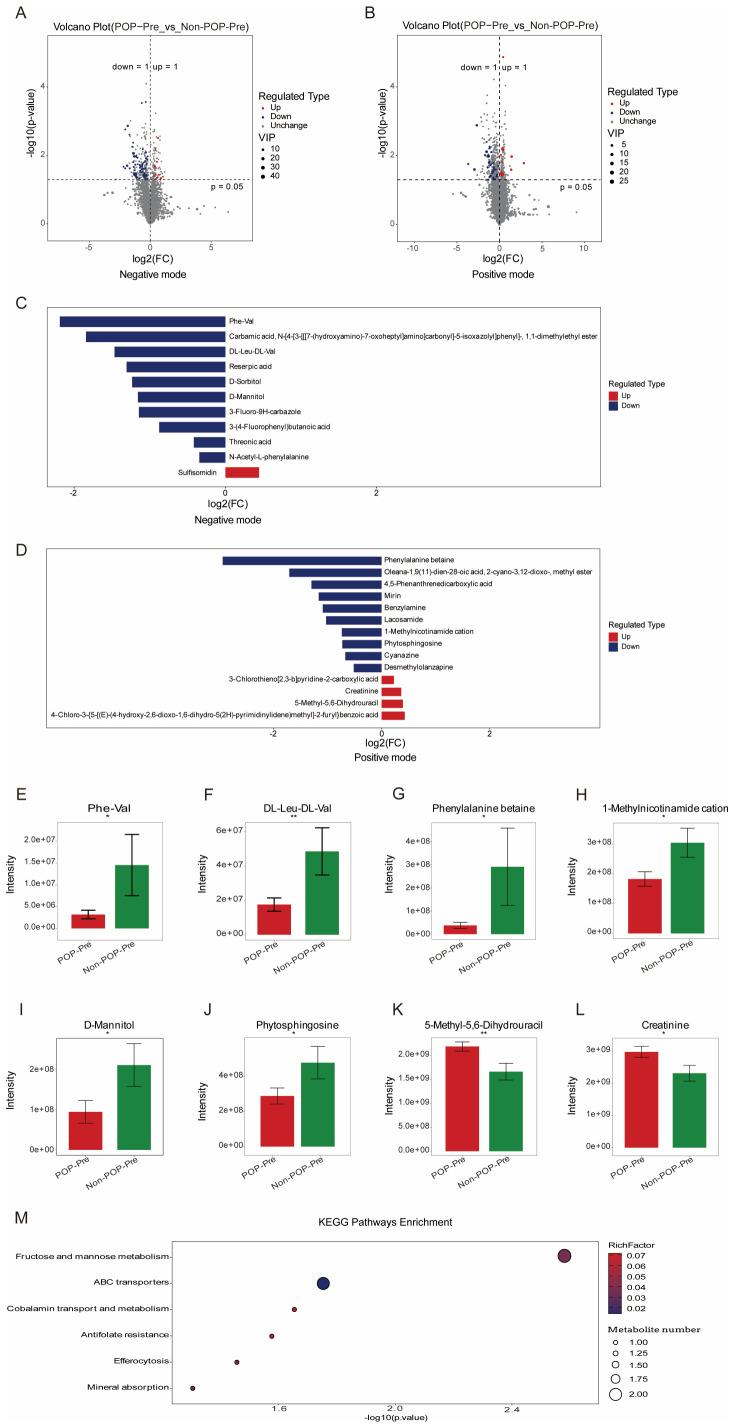
Differential plasma metabolomic profiles between POP-pre and non-POP-pre subgroups. **(A, B)** Volcano plots of differential metabolites in negative **(A)** and positive **(B)** ionization modes. **(C, D)** Butterfly plots displaying significant metabolites in negative **(C)** and positive **(D)** modes. **(E–L)** Relative abundance of key differential metabolites across subgroups. *p<0.05; **p<0.01. **(M)** KEGG pathway enrichment analysis of differential metabolites. The x-axis represents -log10(p-value) and the y-axis indicates enriched pathways. Blue dots represent the Rich Factor (ratio of differential to total metabolites in a pathway). Numbers indicate the count of differential metabolites involved. POP-Pre, preoperative of postoperative pneumonia; Non-POP-Pre, preoperative of postoperative non-pneumonia.

Using non-targeted metabolomics analysis, a total of 948 metabolites were identified in plasma samples collected from 50 subjects before and after surgery, primarily covering chemical classifications such as Lipids_and_lipid_like_molecules (255, 26.34%), Organic_acids_and_derivatives (212, 21.90%), Organoheterocyclic_compounds (207, 21.38%), Benzenoids (127, 13.12%), and Organic_oxygen_compounds (51, 5.27%). Volcano plots demonstrated that the POP-Pre subgroup had more downregulated metabolites compared to the Non-POP-Pre subgroup (**Figures 5A–D**). Specifically, Phe-Val was significantly downregulated in negative mode (Fold Change ≈ 0.22). In positive mode, Phenylalanine betaine (Fold Change ≈ 0.13), 1-Methylnicotinamide (1-MNA), and Phytosphingosine were also significantly decreased (**Figures 5E, F**). Pathway enrichment analysis showed that differential metabolites were involved in Fructose and mannose metabolism, ABC transporters, and Cobalamin transport and metabolism (**Figure 5M**). Notably, “Fructose and mannose metabolism” showed the highest significance (-*log_10_(p*-value)), indicating substantial preoperative carbohydrate metabolic dysfunction in POP patients.

### 3.5 Multi-omics correlation mechanism

**Figure 6:**
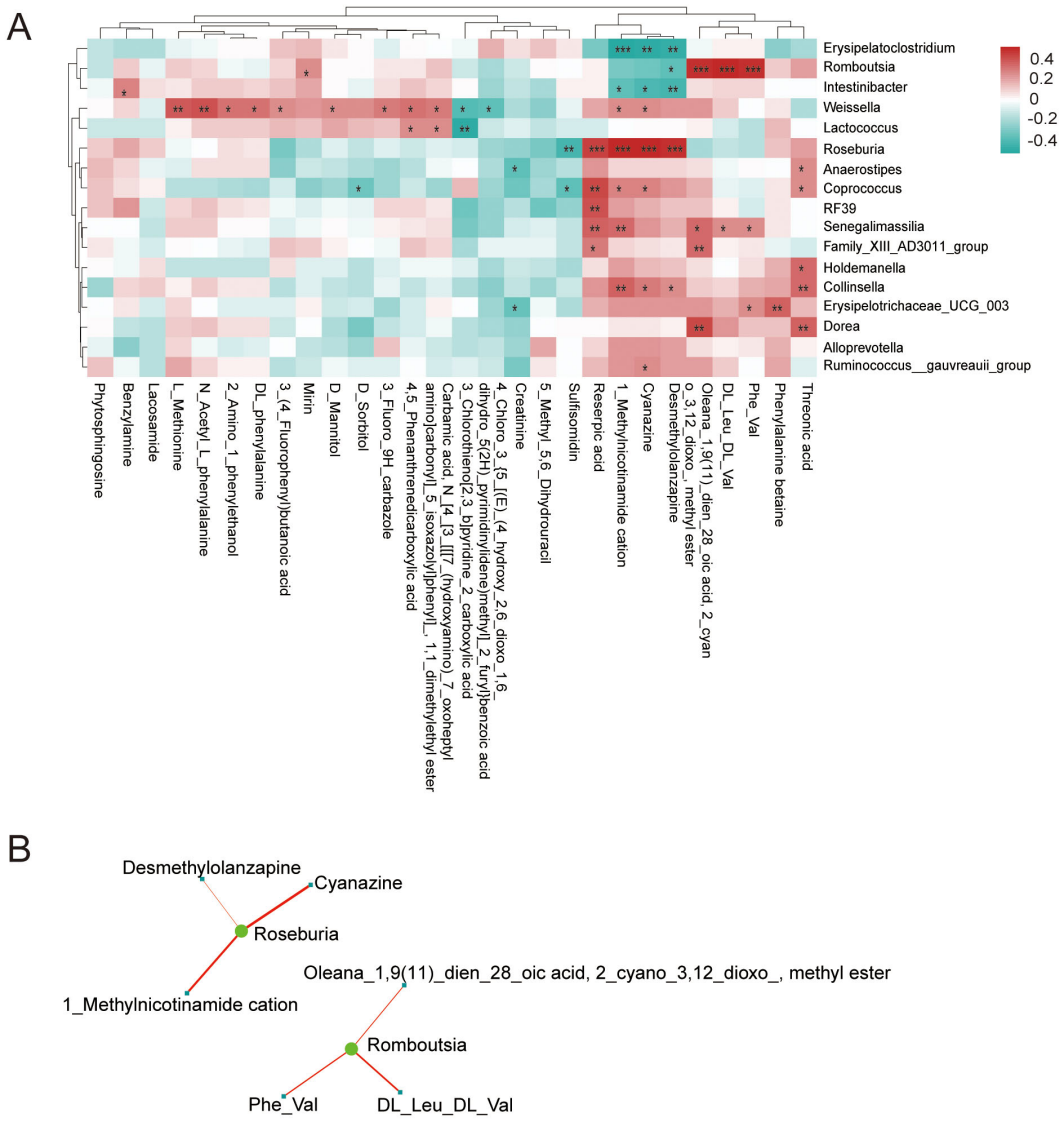
Correlation between differential microbiota and plasma metabolites. **(A)** Heatmap of Spearman correlation coefficients between significant microbial genera and metabolites. **(B)** Network diagram illustrating significant associations. Red lines: positive correlation; Blue lines: negative correlation. *p<0.05; **p<0.01, ***p<0.001.

To further explore the potential functional associations between differential microbiota and plasma metabolites in the POP-Pre and Non-POP-Pre subgroups, Spearman correlation analysis was performed on 17 significantly altered microbial genera and 28 differential metabolites. The correlation heatmap (**Figure 6A**) intuitively illustrates the interactive signatures between these variables. Subsequently, a correlation network (**Figure 6B**) was constructed to visualize the robust associations (|*r*| > 0.5, *p* < 0.05) among microorganisms, metabolites, and their related pathways. The results revealed a clustered network architecture centered on *Roseburia* and *Romboutsia* as core nodes, identifying a total of six significantly positively correlated microbe-metabolite modules. *Roseburia* and 1-MNA indicate disruption of the anti-inflammatory axis. *Romboutsia* and Phe-Val, Leu-Val suggest disruption of the nutritional axis.

### 3.6 Metabolite-based models for clinical prediction

**Figure 7:**
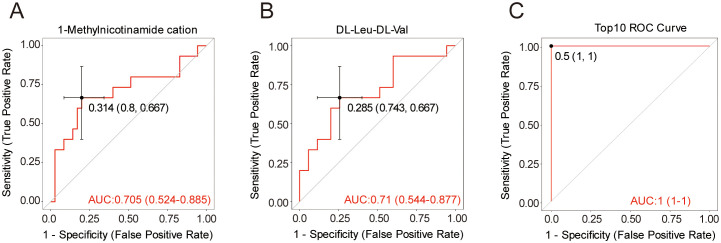
ROC analysis of metabolites for predicting postoperative pulmonary infection. **(A, B)** Predictive performance (AUC) of individual metabolites: 1-Methylnicotinamide cation **(A)** and DL-Leu-DL-Val. **(B)**. **(C)** Combined ROC curve of the Top 10 differential metabolites.

To assess clinical utility, ROC curve analysis was performed in the POP-Pre and Non-POP-Pre subgroups. All 28 differential metabolites yielded Area Under the Curve (AUC) values > 0.6 (**Supplementary Table 2**). Specifically, DL-Leu-DL-Val and 1-MNA cation achieved AUCs of 0.71 and 0.70, respectively (**Figures 7A, B**). Based on univariate AUC ranking, a combined ROC model was developed using the top 10 metabolites, including 4-Chloro-3-{5-[(E)-(4-hydroxy-2,6-dioxo-1,6-dihydro-5(2H)-pyrimidinylidene)methyl]-2-furyl}benzoic acid, 4,5-Phenanthrenedicarboxylic acid, Carbamic acid, N-[4-[3-[[[7-(hydroxyamino)-7-oxoheptyl]amino]carbonyl]-5-isoxazolyl]phenyl]-, 1,1-dimethylethyl ester, Mirin, Benzylamine, L-Methionine, N-Acetyl-L-phenylalanine, DL-Leu-DL-Val, 1-Methylnicotinamide cation, and 3-(4-Fluorophenyl)butanoic acid. This model achieved excellent performance with an AUC of 1.0, demonstrating superior classification and predictive capability (**Figure 7C**).

## 4 Discussion

The present study provides a comprehensive longitudinal characterization of the gut microbiota and plasma metabolome in patients undergoing craniotomy for intracranial space-occupying lesions. Our multi-omics integrated analysis demonstrates that preoperative dysbiosis (manifested as depletion of butyrate-producing bacteria) and the subsequent systemic metabolic homeostasis disruption constitute the biological basis for postoperative infection susceptibility in patients.

### 4.1 Disruption of the gut-lung anti-inflammatory axis: *Roseburia* and 1-MNA

The gut microbiota is not confined to the intestinal tract but also exerts remote regulation on pulmonary immune homeostasis through the secretion of metabolites (6, 11, 12). This study revealed that although intracranial space-occupying lesions and surgical trauma were associated with certain shifts in the gut microbiota, no statistically significant differences in α-diversity were found between the groups (*p* > 0.05). Nevertheless, we identified a significant preoperative reduction in *Roseburia*—a predominant butyrate-producing genus essential for intestinal barrier integrity and regulatory T cell (Treg) differentiation (13) —among patients who developed POP. Our findings suggest that this depletion is positively correlated with the deficiency of 1-MNA (**Figure 6A**), an anti-inflammatory metabolite that modulates macrophage activity and inhibits pro-inflammatory cytokines (14, 15). We hypothesize that the disruption of this “anti-inflammatory axis” contributes to a “pro-inflammatory susceptible” state in the lungs. Consequently, during surgical stress (e.g., intubation), the host might lack sufficient immune buffering mechanisms, potentially linking these preoperative signatures to the development of POP.

### 4.2 Bioenergetic imbalance: carbohydrate metabolism and immune readiness

KEGG pathway analysis suggested that Fructose and mannose metabolism was the most significantly altered preoperative pathway (**Figure 5M**), offering a novel perspective on the potential immunometabolic basis of POP. Beyond providing cellular energy, carbohydrate metabolism is fundamentally linked to innate immune activation (16). Mannose, in particular, is essential for cosy immune receptors glycosylation and modulates the phagocytic capacity of alveolar macrophages (17). The significant downregulation of this pathway in the POP-Pre subgroup is consistent with a state of “metabolic exhaustion” before surgery. During the high-stress period following craniotomy, innate immune cells must undergo rapid metabolic reprogramming to fuel their antimicrobial functions. A preoperative defect in these bioenergetic pathways is associated with potential “energy failure” during early host defense, which may allow opportunistic pathogens to bypass immune surveillance and establish infection.

### 4.3 Nutritional reserve deficits and tissue repair capacity

Our metabolomic profiling observed a marked reduction in essential amino acid-related dipeptides, (e.g., Phe-Val and Leu-Val) (**Figures 5E, F**) and significant alterations in the ABC transporters pathway with the POP-Pre subgroup (**Figure 5M**). These findings are consistent with a preoperative “micro-nutritional deficit.”

Given that ABC transporters are vital for nutrient uptake (18), their negative correlation with *Romboutsia* enrichment (**Figure 6**) suggests a potential link between microbial overgrowth and impaired nutrient absorption. Following surgical trauma, the rapid repair of the respiratory epithelium and alveolar-capillary barrier requires a steady supply of protein and lipid precursors. The preoperative depletion of these nutritional reserves is associated with reduced efficiency of tissue repair, potentially rendering making the lungs more vulnerable to persistent pathogen colonization.

### 4.4 Clinical implications, predictive potential, and limitations

Despite the clinical relevance of our findings, several limitations must be acknowledged. First, our predictive model’s AUC of 1.0 likely reflects overfitting due to the small sample size (N = 50) and group imbalance (n=35 vs n=15). Lacking an independent validation cohort, these results should be viewed as a preliminary discovery requiring verification in larger, multicenter studies. Second, while our core predictive markers were identified in antibiotic-free preoperative samples, the higher rate of therapeutic antibiotic use in the POP group postoperatively (*p* = 0.015) may have confounded the microbial evolution in later samples. Future research using germ-free models is needed to isolate these antibiotic-induced shifts from the host’s inherent signatures. Moreover, the observational nature of this study precludes the establishment of causality. While the *Roseburia*-1-MNA correlations are robust, they do not confirm whether microbial depletion directly drives pulmonary susceptibility. Further studies utilizing fecal microbiota transplantation (FMT) or probiotic supplementation in animal models are required to verify these mechanistic links and their therapeutic potential.

## 5 Conclusion

Through a prospective cohort study of 50 patients with intracranial space-occupying lesions, this study systematically analyzed preoperative and postoperative gut microbial and peripheral metabolic signatures using 16S rDNA sequencing and untargeted metabolomics. The results suggest that preoperative dysbiosis, characterized by the depletion of *Roseburia*, along with a key metabolic signature composed of molecules such as 1-MNA, is associated with the risk of POP. This finding underscores the potential clinical value of preoperative metabolic assessment in identifying high-risk individuals and provides a scientific basis for further exploring early precision intervention during the perioperative period.

## Data Availability

The raw sequence data reported in this paper have been deposited in the Genome Sequence Archive (Genomics, Proteomics & Bioinformatics 2025) in National Genomics Data Center (Nucleic Acids Res 2026), China National Center for Bioinformation/Beijing Institute of Genomics, Chinese Academy of Sciences (GSA: CRA043053) that are publicly accessible at https://ngdc.cncb.ac.cn/gsa.
