# Supplementary material for: Preoperative gut microbiota depletion and metabolomic signatures predict postoperative pneumonia in patients with intracranial space-occupying lesions: a multi-omics prospective cohort study
Source: Front Immunol. 2026 Jun 16;17:1839465. doi: 10.3389/fimmu.2026.1839465 (PMC13314423; doi:10.3389/fimmu.2026.1839465)
Supplement: Supplementary file 1 [file Table1.docx]

**Supplementary Table 1 Clinical baseline characteristics of the study population.**

| Characteristics | POP Group (n=35) | Non-POP Group (n=15) | *p*-value |
| --- | --- | --- | --- |
| Age (years) | 52.03±11.76 | 45.47±12.97 | 0.470 |
| Gender (Male, %) | 20 (57.1%) | 6 (40.0%) | 0.387 |
| BMI (kg/m^2^) | 24.92±4.32 | 24.29±3.09 | 0.290 |
| Height (cm) | 161.85 ± 8.21 | 163.27 ± 9.01 | 0.719 |
| Weight (kg) | 65.27 ± 12.20 | 65.13 ± 11.96 | 0.985 |
| Smoking history (yes) | 3 (8.6%) | 4 (26.7%) | 0.176 |
| Surgical duration (min) | 274.54 ± 106.87 | 276.13± 169.71 | 0.436 |
| ICU admission duration (h) | 15 (0-60) | 0 (0-14) | 0.070 |
| Ventilator use duration (h) | 15 (3-23) | 4 (3.17) | 0.172 |
| Postoperative therapeutic antibiotics (yes) | 23 (65.7%) | 4 (26.7%) | 0.015 |
| Postoperative diagnosis of pneumonia (h) | 16.73±5.48 | - | - |

Data are presented as mean ± standard deviation, median (p25-p27),or frequency (percentage).

POP: Postoperative Pneumonia; Non-POP: Non-postoperative Pneumonia; -: uninvolved.

**Supplementary Table 2 ROC curve analysis of individual metabolites in the differential metabolites.**

| **Metabolite Name** | **AUC** |
| --- | --- |
| **4-Chloro-3-{5-[(E)-(4-hydroxy-2,6-dioxo-1,6-dihydro-5(2H)-pyrimidinylidene)methyl]-2-furyl}benzoic acid** | 0.87 |
| **4,5-Phenanthrenedicarboxylic acid** | 0.77 |
| **Carbamic acid, N-[4-[3-[[[7-(hydroxyamino)-7-oxoheptyl]amino]carbonyl]-5-isoxazolyl]phenyl]-, 1,1-dimethylethyl ester** | 0.75 |
| **Mirin** | 0.75 |
| **Benzylamine** | 0.73 |
| **L-Methionine** | 0.72 |
| **N-Acetyl-L-phenylalanine** | 0.71 |
| **DL-Leu-DL-Val** | 0.71 |
| **1-Methylnicotinamide cation** | 0.70 |
| **3-(4-Fluorophenyl)butanoic acid** | 0.70 |
| 3-Chlorothieno[2,3-b]pyridine-2-carboxylic acid | 0.70 |
| Creatinine | 0.70 |
| 5-Methyl-5,6-Dihydrouracil | 0.69 |
| Sulfisomidin | 0.69 |
| Reserpic acid | 0.68 |
| Cyanazine | 0.68 |
| 2-Amino-1-phenylethanol | 0.68 |
| DL-phenylalanine | 0.67 |
| D-Mannitol | 0.67 |
| Oleana-1,9(11)-dien-28-oic acid, 2-cyano-3,12-dioxo-, methyl ester | 0.67 |
| Desmethylolanzapine | 0.66 |
| D-Sorbitol | 0.66 |
| Threonic acid | 0.66 |
| 3-Fluoro-9H-carbazole | 0.65 |
| Lacosamide | 0.65 |
| Phytosphingosine | 0.64 |
| Phe-Val | 0.62 |
| Phenylalanine betaine | 0.62 |
